# RNA-seq Analysis of Irrigated vs. Water Stressed Transcriptomes of *Zea mays* Cultivar Z59

**DOI:** 10.3389/fpls.2016.00239

**Published:** 2016-03-01

**Authors:** B. Divya Bhanu, Kandasamy Ulaganathan, Arun K. Shanker, S. Desai

**Affiliations:** ^1^Centre for Plant Molecular Biology, Osmania UniversityHyderabad, India; ^2^Central Research Institute for Dryland AgricultureHyderabad, India

**Keywords:** RNA-seq, transcriptome, *Zea mays*, Z59, water stress, drought

## Introduction

Maize (*Zea mays*) is one of the important food crops of India with other potential uses in preparation of livestock feed and biofuel. But production of maize in India is low compared to top maize producing countries due to abiotic stresses (Ranum et al., 2014). Among abiotic stresses affecting maize production in India, drought is the major limiting factor. In spite of extensive research on drought tolerance in maize no tangible achievements could be made due to the extreme complexity associated with drought tolerance (Ashraf, 2010). Availability of the maize genome and high throughput genomic methods have provided new tools for addressing the complexity associated with drought tolerance (Zivy et al., 2015). We have chosen the drought tolerant genotype Z59 and are developing transcriptomic resources for better understanding of drought tolerance of this cultivar. As part of this work, we have sequenced the irrigated and water stressed transcriptomes of Z59 cultivar and the dataset is reported here.

## Materials and methods

### Plant material used

Seedlings of *Zea mays* Z59 cultivar were grown in separate pots of uniform soil under greenhouse conditions. Plants were grown under well-watered conditions until they reached the early reproductive stage. Drought was imposed at early reproductive-phase by withholding irrigation for half of the plants. Both control plants (irrigated) and stressed plants (50% relative water content) were used for transcriptome sequencing.

### Library preparation and transcriptome sequencing

Leaf tissue (lamina, topmost leaf) was collected from control- and drought-treated plants and stored in “RNAlater” solution (Thermo Fisher Scientific) at −80°C. RNA isolation was carried out using the RNeasy Plant kit (Quiagen, 2015). The leaf samples were ground to fine powder using liquid nitrogen in a mortar and pestle and subsequent isolation steps were as per the instructions provided in the RNeasy Plant Kit. The concentration and purity of the RNA extracted was evaluated using the NanoDrop Spectrophotometer (Thermo Scientific- 1000). The integrity of the extracted RNA was analyzed on the Bioanalyzer (Agilent, 2100). Irrigated and water stressed RNA samples with 6.9 and 7.8 RNA integrity numbers, respectively were used for library preparation.

Library preparation was performed using Illumina TruSeq RNA library protocol developed by Illumina Technologies (San Diego, CA). 1 ug of total RNA was subjected to PolyA purification of mRNA. Purified mRNA was fragmented for 8 minutes at elevated temperature (94°C) in the presence of divalent cations and reverse transcribed with SuperScript III Reverse Transcriptase by priming with random hexamers. Second strand cDNA was synthesized in the presence of DNA polymerase I and RnaseH. The cDNA was cleaned up using HighPrep PCR (MAGBIO, Cat# AC-60050). Illumina adapters were ligated to the cDNA molecules after end repair and addition of A base. SPRI (solid-phase reversible immobilization, Beckman Coulter) cleanup was performed after ligation. The library was amplified using 8 cycles of PCR for enrichment of adapter ligated fragments. The prepared library was quantified using Qubit and validated for quality by running an aliquot (1 μl) on High Sensitivity DNA Kit (Agilent) which showed expected fragment distribution in the range of ~250–500 bp. The effective sequencing insert size was ~130–380 bp; the inserts were flanked by adapters whose combined size was ~130 bp. Transcriptome sequencing was carried out with the Illumina-Nextseq500 system (Illumina, San Diego, CA).

### Preprocessing and assembly of transcripts

The preprocessing of the reads was performed with FastQC (Andrews, 2010) and the adapters were removed by using Cutadapt tool (Martin, 2011). *De novo* transcriptome assembly was performed with Velvet and Oases software (Zerbino and Birney, 2008; Schulz et al., 2012).

## Results

Transcriptome sequencing resulted in 17369114 and 21543071 reads in irrigated and water stressed samples, respectively. The read length after trimming was found to be 76 base pairs. *De novo* assembly of the reads generated a total of 31399 and 30157 transcripts in irrigated and water stressed samples of Z59, respectively. The summary of the transcriptomes are listed in Table 1. Further comparative analysis of irrigated and drought stressed transcriptomes is in progress to identify genes differentially expressed during drought stress and also to identify differential alternate splicing of transcripts during drought stress.

**Table 1:** ***Zea mays Z59* transcriptome statistics**.

**Feature**	**Irrigated**	**Water stressed**
NCBI project ID	PRJNA300830	PRJNA300830
NCBI biosample ID	SAMN04229490	SAMN04229491
NCBI SRA accession number	SRP065617	SRP065617
NCBI transcriptome accession number	GECR00000000	GECS00000000
Sequence type	Illumina Nextseq 500	Illumina Nextseq 500
Total number of reads	34738228	43086142
Read length	76	76
No. of *De novo* transcripts	31399	30157

### Direct link to deposited data and information to users

The dataset submitted to NCBI include the assembled transcriptome sequences of irrigated and water stressed plants of *Zea mays* cultivar Z59 in Fasta format and the raw reads. The assembled transcriptome sequences of the irrigated and water stressed plants of *Zea mays* cultivar Z59 can be accessed at NCBI with the following accession numbers: GECR00000000 (irrigated); GECS00000000 (water stressed). Raw reads of both transcriptomes can be accessed with the following NCBI accession number: SRP065617. Users can download and use the data freely for research purpose only with acknowledgment to us and quoting this paper as reference to the data. Comparative analysis of Z59 (drought tolerant cultivar) transcriptomes with other transcriptomes made from other drought tolerant and susceptible maize cultivars can be used for effective identification of genes associated with drought tolerance in maize.

## Author contributions

The work was planned jointly and executed by BD and KU. AS and SD were involved in selection and growth of the plant material.

## Funding

The work was carried out with financial assistance from Department of Science and Technology, Ministry of Science and Technology, Government of India, New Delhi.

### Conflict of interest statement

The authors declare that the research was conducted in the absence of any commercial or financial relationships that could be construed as a potential conflict of interest. The reviewer, RD, and handling Editor declared their shared affiliation, and the handling Editor states that the process nevertheless met the standards of a fair and objective review.
